# SGLT2 inhibitor ipragliflozin alone and combined with pioglitazone prevents progression of nonalcoholic steatohepatitis in a type 2 diabetes rodent model

**DOI:** 10.14814/phy2.14286

**Published:** 2019-11-28

**Authors:** Atsuo Tahara, Toshiyuki Takasu

**Affiliations:** ^1^ Drug Discovery Research Astellas Pharma Inc. Ibaraki Japan

**Keywords:** ipragliflozin, NASH, SGLT2, type 2 diabetes

## Abstract

Nonalcoholic steatohepatitis (NASH) has become the most common cause of chronic liver disease worldwide in recent years. The pathogenesis of NASH is closely linked to metabolic diseases such as insulin resistance, obesity, dyslipidemia, and type 2 diabetes. However, there is currently no pharmacological agent for preventing the progression of NASH. Sodium–glucose cotransporter (SGLT) 2 inhibitors increase urinary glucose excretion by inhibiting renal glucose reabsorption, and improve various pathological conditions of type 2 diabetes, including insulin resistance. In the present study, we examined the effects of ipragliflozin, a SGLT2‐selective inhibitor, alone and in combination with pioglitazone on NASH in high‐fat diet‐fed KK/A^y^ type 2 diabetic mice. Type 2 diabetic mice with NASH exhibited steatosis, inflammation, and fibrosis in the liver as well as hyperglycemia, insulin resistance, and obesity, features that are observed in human NASH. Four‐week repeated administration of ipragliflozin (0.1–3 mg/kg) led to significant improvements in hyperglycemia, insulin resistance, and obesity in addition to hyperlipidemia and liver injury including hepatic steatosis and fibrosis. Moreover, ipragliflozin reduced inflammation and oxidative stress in the liver. Repeated administration of pioglitazone (3–30 mg/kg) also significantly improved various parameters of diabetes and NASH, excluding obesity. Furthermore, combined treatment comprising ipragliflozin (1 mg/kg) and pioglitazone (10 mg/kg) additively improved these parameters. These findings indicate that the SGLT2‐selective inhibitor ipragliflozin improves hyperglycemia as well as NASH in type 2 diabetic mice. Therefore, treatment with ipragliflozin monotherapy or coadministered with pioglitazone is expected to be a potential therapeutic option for the treatment of type 2 diabetes with NASH.

## Abstract 2

Ipragliflozin monotherapy or coadministered with pioglitazone prevented the development of pathological features of NASH, suggesting that it is a potential new therapeutic strategy for NASH associated with type 2 diabetes.

## Graphical abstract

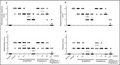

## 1 INTRODUCTION

In recent decades, type 2 diabetes has become increasingly prevalent. The incidence of nonalcoholic fatty liver disease (NAFLD) has also increased worldwide, becoming the most common liver disorder in the industrialized world (Wattacheril, Issa, & Sanyal, 2018). NAFLD includes a wide spectrum of histological abnormalities ranging from fat accumulation in hepatocytes with no associated inflammation of fibrosis (simple steatosis) or steatosis with inflammation to nonalcoholic steatohepatitis (NASH), comprising macrovesicular steatosis, lobular inflammation, balloon degeneration of hepatocytes, and fibrosis, which progresses to cirrhosis and hepatocellular carcinoma (McCullough, 2006). NASH is linked to metabolic diseases such as insulin resistance, obesity, and type 2 diabetes, and can be viewed as a hepatic phenotype for metabolic syndrome (Marchesini et al., 2003). The preferred first‐line therapeutic approach for NASH is the introduction of lifestyle interventions to reduce a patient's weight by >7%, such as changes to diet and lifestyle habits including regular exercise (Mavrogiannaki & Migdalis, 2013). However, because these lifestyle interventions are often hard to execute and uphold, their effectiveness is limited. Therefore, there is a need for new therapeutic approaches for the management of NASH. While a number of recent studies have reported therapeutic interventions including pioglitazone and metformin for NASH (Barb, Portillo‐Sanchez, & Cusi, 2016; Boettcher, Csako, Pucino, Wesley, & Loomba, 2012; Li, Liu, Wang, Wang, & Chen, 2013; Portillo‐Sanchez & Cusi, 2016), the level of evidence remains low. At present, the ideal treatment for NASH has yet to be identified.

Sodium–glucose cotransporter 2 (SGLT2) inhibitors, which inhibit the reabsorption of filtered glucose in the kidneys to increase urinary glucose excretion, were recently developed and proposed as novel anti‐hyperglycemic agents for treating type 2 diabetes (Chao, 2014). In addition, evidence indicates that not only do SGLT2 inhibitors improve hyperglycemia but they also improve insulin resistance, hyperlipidemia, and obesity (Kurosaki & Ogasawara, 2013; Takase, Nakamura, Miyoshi, Yamamoto, & Atsumi, 2017). In addition, several clinical studies have reported that SGLT2 inhibitors have favorable effects on NAFLD/NASH in patients with type 2 diabetes (Ohta et al., 2017; Scheen, 2019; Yamauchi et al., 2019). These findings suggest that SGLT2 inhibitors may have a beneficial effect on a wide range of pathologies caused by metabolic diseases including NASH. We have investigated the therapeutic effects of the SGLT2 inhibitor ipragliflozin on NASH using a number of animal models. We found that ipragliflozin significantly attenuated various diabetic features such as hyperglycemia, insulin resistance, and obesity, and NASH‐related features such as liver inflammation and steatosis in KK/A^y^ or high‐fat diet‐ and streptozotocin–nicotinamide‐induced type 2 diabetic mice (Tahara et al., 2013; Tahara, Takasu, Yokono, Imamura, & Kurosaki, 2017). While these mice exhibit obesity, insulin resistance, and hepatic steatosis, they do not exhibit hepatic fibrosis, which is observed in human NASH. In another study, we found that ipragliflozin exerted a prophylactic effect on liver fibrosis in choline‐deficient L‐amino acid‐defined (CDAA) diet‐induced NASH rats (Hayashizaki‐Someya et al., 2015). While this model exhibits marked increases in plasma aminotransferase levels and hepatic histological changes such as steatosis, inflammation, necrosis, and fibrosis, it does not exhibit obesity and insulin resistance, which are typical features of human NASH. Therefore, a more optimal animal model that exhibits pathophysiological features similar to human NASH is needed to investigate the therapeutic effects of drugs of interest on NASH.

In this study, we examined the effects of ipragliflozin on a variety of diabetic features and the progression of NASH in high‐fat diet‐fed type 2 diabetic mice with hyperglycemia, insulin resistance, obesity, and liver injury including fibrosis. We also compared the effects of ipragliflozin and pioglitazone, and examined the effects of combination therapy with ipragliflozin and pioglitazone.

## 2 MATERIALS AND METHODS

### 2.1 Materials and animals

Ipragliflozin and pioglitazone were synthesized at Astellas Pharma Inc. (Ibaraki, Japan) and suspended in 0.5% methylcellulose solution and administered orally through a stomach tube. Drugs were administered once daily at night (19:00–20:00) and doses were expressed as the free base form. Male C57BL/6 (normal) and KK/A^y^ type 2 diabetic mice were purchased from Charles River Laboratories Japan, Inc. (Tokyo, Japan) and CLEA Japan (Kanagawa, Japan), respectively, at 6 weeks of age and fed either a normal chow diet consisting (as a percentage of total calories [kcal]) of 11% fat, 69% carbohydrate, and 20% protein (total caloric energy = 3.7 kcal/g; D12337; Research Diets, Inc., New Brunswick, NJ, USA) or a high‐fat diet consisting of 45% fat, 35% carbohydrate, and 20% protein (total caloric energy = 4.73 kcal/g; D12451; Research Diets, Inc.). Diabetic mice at 7 weeks of age were grouped such as to ensure uniform mean blood glucose levels among groups. All animals were housed under conventional conditions with controlled temperature, humidity, and light (12‐h light–darkcycle) and were provided food and water ad libitum. All animal experimental procedures were approved by the Institutional Animal Care and Use Committee of Tsukuba Research Center of Astellas Pharma Inc., which is accredited by the Association for Assessment and Accreditation of Laboratory Animal Care (AAALAC) International.

### 2.2 Repeated administration study

Vehicle, ipragliflozin (0.1–3 mg/kg), pioglitazone (3–30 mg/kg), or ipragliflozin (1 mg/kg) + pioglitazone (10 mg/kg) was orally administered to diabetic mice with NASH for 4 weeks, and body weight was measured weekly. Daily food consumption was calculated based on the weekly difference between the food remaining and food supplied. After drug administration at night on Day 14, blood samples for the evaluation of blood glucose and plasma insulin levels were obtained from the tail vein at each sampling point for 24 hr.

An oral glucose tolerance test (OGTT) was performed at Week 3. After drug administration at night on Day 22, mice were fasted for half a day. The following morning, blood was sampled from the tail vein and used to examine fasting blood glucose and plasma insulin levels. A glucose solution (2 g/kg) was orally administered, and blood sampling was conducted for 2 hr. After drug administration at night on Day 26, mice were transferred to metabolic cages and spontaneously voided urine was collected for 24 hr. On the morning after the final drug administration (Day 28), blood samples were collected under nonfasting conditions, and liver and visceral (epididymal, perirenal, and mesenteric) fat tissues were isolated under isoflurane anesthesia.

### 2.3 Biochemical tests

Blood and urine glucose concentrations were measured using the Glucose CII test reagent (Wako Pure Chemical Industries, Ltd., Osaka, Japan). Hemoglobin A_1c_ (HbA_1c_) levels were measured using a DCA2000 System (Bayer Medical, Tokyo, Japan). Plasma insulin levels were measured using an enzyme‐linked immunosorbent assay (ELISA) kit (Morinaga Institute of Biological Science, Inc., Kanagawa, Japan). Plasma lipid concentrations and hepatic lipid contents were measured in reference to a previously described method (Tahara, Matsuyama‐Yokono, & Shibasaki, 2011). Levels of aminotransferases—alanine amino transferase (ALT) and aspartate amino transferase (AST)—were measured using the Transaminase CII test reagent (Wako Pure Chemical Industries, Ltd.). Concentrations of plasma and liver cytokines, interleukin 6 (IL‐6), tumor necrosis factor α (TNF‐α), and monocyte chemotactic protein‐1 (MCP‐1), and c‐reactive protein (CRP) were measured in accordance with a previously reported method (Ji et al., 2011) using commercial ELISA kits (R&D Systems Inc., Minneapolis, MN, USA). Concentrations of plasma adiponectin, leptin, and fibroblast growth factor 21 (FGF‐21) were measured using commercial ELISA kits (R&D Systems Inc.). Levels of oxidative stress biomarkers (thiobarbituric acid reactive substances [TBARS] and protein carbonyl) were measured with reference to a previously described method (Nakhaee, Bokaeian, Saravani, Farhangi, & Akbarzadeh, 2009) using commercial assay kits (Cayman Chemical Company, Ann Arbor, MI, USA).

### 2.4 Histopathology

Preparation of specimens and histopathological examination were performed at CMIC Bioresearch Center Co., Ltd. (Yamanashi, Japan). Sagittal slices of liver fixed in 10% neutral buffered formalin were embedded in paraffin and cut into sections of 2 μm thickness for morphological study. The sections were stained with hematoxylin and eosin (H&E) and sirius red. All tissue samples were evaluated by an independent investigator blinded to grouping data. Microvesicular steatosis and hepatocellular hypertrophy were evaluated based on the percentage of area affected (0: <5%, 1:5%–33%, 2:34%–66%, 3: >66%). Inflammation was evaluated by counting the number of inflammatory foci per field (0: normal, <0.5 foci; 1: slight, 0.5–1 foci; 2: moderate, 1–2 foci; 3: severe, >2 foci). Hepatic fibrosis was evaluated using a semi‐quantitative scoring system (0: normal, 1: slight, 2: moderate, 3: severe) (Uehara et al., 1993).

### 2.5 Statistical analysis

Data are expressed as the mean ± standard error of the mean (*SEM*) or individual data and median. Areas under the curve (AUCs) were calculated from blood glucose and plasma insulin concentrations measured over time. The Matsuda index was calculated using the following formula: 10,000/square root of [(fasting blood glucose × fasting plasma insulin) × (mean blood glucose × mean plasma insulin during OGTT)], and the disposition index was calculated using the following formula: (plasma insulin AUC/blood glucose AUC during OGTT) × Matsuda index (Matsuda & DeFronzo, 1999). Differences between normal and diabetic vehicle groups were assessed for significance using Student's *t*‐test, while those between the vehicle‐ and drug‐treated groups were assessed using Dunnett's multiple comparisons test. For the histopathological scores, Mann–Whitney *U* test was used to analyze differences between normal and diabetic vehicle groups, while Dunn's multiple comparisons test was used for comparisons between vehicle‐ and drug‐treated groups. A value of *p* < .05 was considered statistically significant. Statistical and data analyses were conducted using GraphPad Prism 5 (GraphPad Software, La Jolla, CA, USA).

## 3 RESULTS

**Figure 1:**
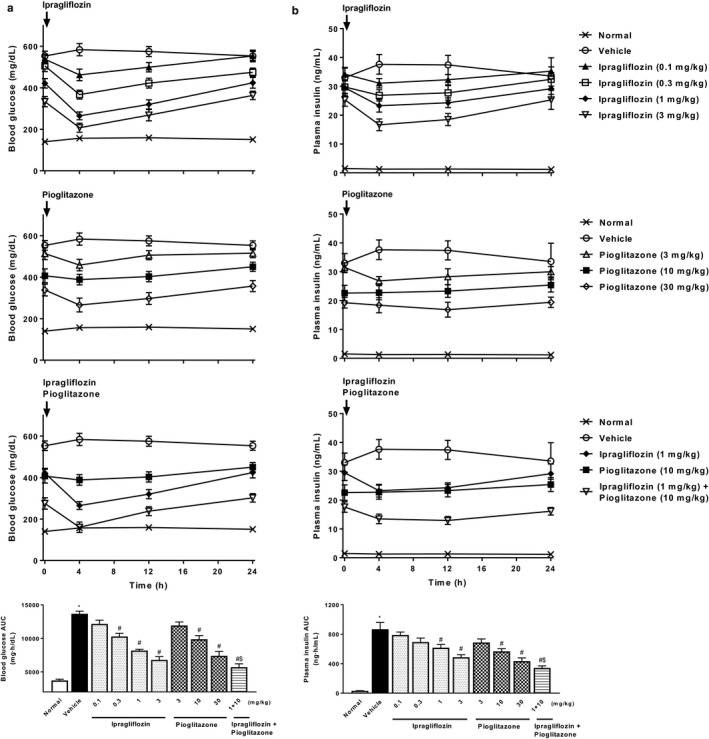
Effects of single or combined treatment with ipragliflozin and pioglitazone on blood glucose and plasma insulin levels in type 2 diabetic mice with NASH. Time course of changes in (a) blood glucose and (b) plasma insulin levels, and the area under the blood glucose and plasma insulin concentration–time curve (AUC) for 24 hr. Blood glucose and plasma insulin levels were measured at Week 2 of repeated administration. Values indicate mean ± *SEM* for six animals per group. *p < .05 versus normal group, ^#^ *p* < .05 versus vehicle group, ^$^ *p* < .05 versus pioglitazone (10 mg/kg) group

**Figure 2:**
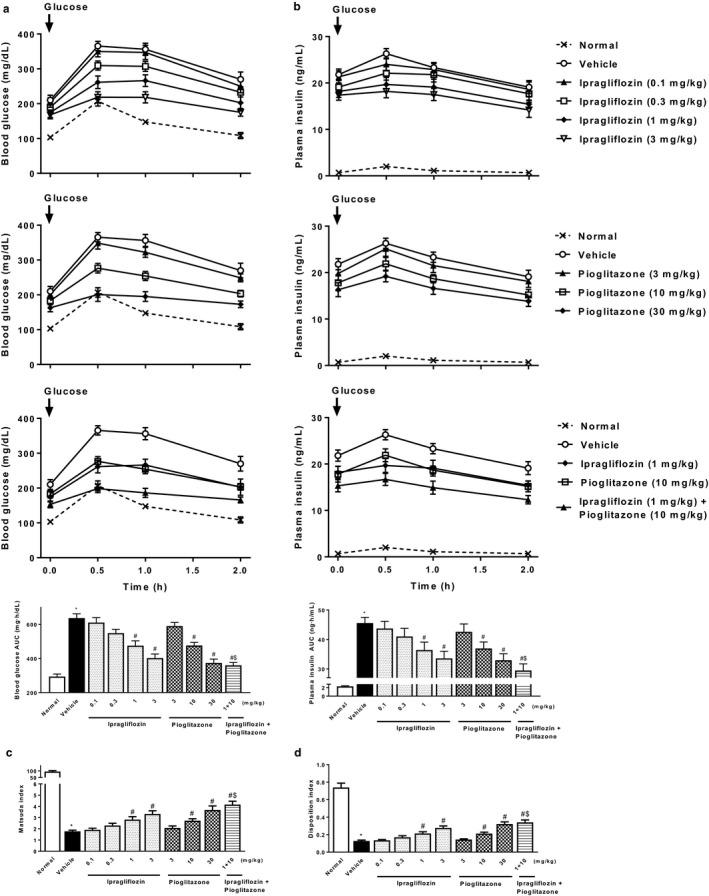
Effects of single or combined treatment with ipragliflozin and pioglitazone on glucose tolerance and insulin resistance in type 2 diabetic mice with NASH. Time course of changes in the (a) blood glucose and (b) plasma insulin levels, and area under the blood glucose and plasma insulin concentration–time curve (AUC) during the oral glucose tolerance test (OGTT). The (c) Matsuda index and (d) disposition index were used as parameters of insulin resistance and secretion, respectively. OGTT was performed at Week 3 of repeated administration. Values indicate mean ± *SEM* for six animals per group. *p < .05 versus normal group, ^#^ *p* < .05 versus vehicle group, ^$^ *p* < .05 versus pioglitazone (10 mg/kg) group

Compared to normal mice, high‐fat diet‐fed diabetic mice exhibited characteristics of type 2 diabetes such as hyperglycemia, hyperinsulinemia, glucose intolerance, hyperphagia, obesity, glycosuria, polyuria, and dyslipidemia (Table 1, Figures 1 and 2). After 2 weeks of repeated administration, ipragliflozin (0.1–3 mg/kg) and pioglitazone (3–30 mg/kg) had dose‐dependently reduced blood glucose and plasma insulin levels (Figure 1), with significant effects observed at doses of 0.3 mg/kg or higher for ipragliflozin and 10 mg/kg or higher for pioglitazone. Reductions in blood glucose and plasma insulin AUCs for 24 hr were comparable between the drugs. Combined treatment comprising ipragliflozin (1 mg/kg) and pioglitazone (10 mg/kg) significantly and additively decreased blood glucose and plasma insulin levels.

**Table 1:** Effects of single or combined treatment with ipragliflozin and pioglitazone in type 2 diabetic mice with NASH

Parameter	Normal	Vehicle	Ipragliflozin				Pioglitazone			Ipragliflozin +
										Pioglitazone
			0.1 mg/kg	0.3 mg/kg	1 mg/kg	3 mg/kg	3 mg/kg	10 mg/kg	30 mg/kg	1 mg/kg +
										10 mg/kg
Body weight (g)	26.4 ± 0.3	52.7 ± 0.7*	51.2 ± 0.8	50.6 ± 0.8	48.2 ± 0.7#	47.1 ± 0.5#	53.2 ± 0.7	56.0 ± 0.9#	57.7 ± 1.2#	51.3 ± 0.7$
Food intake (g/day)	2.83 ± 0.09	5.18 ± 0.13*	4.93 ± 0.19	5.05 ± 0.14	5.11 ± 0.08	5.10 ± 0.08	4.89 ± 0.10	4.99 ± 0.11	5.02 ± 0.15	5.17 ± 0.07
Total visceral fat weight (g)	0.60 ± 0.03	4.09 ± 0.20*	4.16 ± 0.17	3.61 ± 0.14	3.07 ± 0.15#	2.57 ± 0.17#	4.00 ± 0.12	4.56 ± 0.18	5.15 ± 0.13#	3.02 ± 0.15#$
HbA_1c_ (%)	2.95 ± 0.10	10.48 ± 0.51*	9.50 ± 0.35	8.48 ± 0.44#	7.58 ± 0.26#	6.80 ± 0.23#	10.40 ± 0.27	8.65 ± 0.47#	7.97 ± 0.36#	6.80 ± 0.36#$
Blood glucose (mg/dL)	157 ± 4	543 ± 14*	481 ± 37	372 ± 34#	265 ± 21#	202 ± 7#	473 ± 33	355 ± 49#	270 ± 26#	199 ± 11#$
Plasma insulin (ng/mL)	0.9 ± 0.1	39.1 ± 3.6*	35.2 ± 2.7	26.1 ± 2.6#	21.8 ± 1.9#	14.6 ± 1.1#	31.0 ± 2.2	25.3 ± 2.6#	19.6 ± 4.5#	15.5 ± 1.1#$
Plasma triglycerides (mg/dL)	136 ± 14	564 ± 61*	528 ± 33	483 ± 35	392 ± 46#	320 ± 38#	494 ± 22	358 ± 42#	286 ± 27#	193 ± 23#$
Plasma NEFAs (mEq/L)	1.01 ± 0.06	3.54 ± 0.12*	3.42 ± 0.26	3.09 ± 0.34	2.54 ± 0.08#	2.02 ± 0.17#	3.15 ± 0.35	2.53 ± 0.18#	1.72 ± 0.16#	1.86 ± 0.09#$
Plasma cholesterol (mg/dL)	80 ± 13	286 ± 15*	242 ± 14	211 ± 20#	155 ± 7#	121 ± 8#	279 ± 11	210 ± 11#	159 ± 16#	127 ± 15#$
Urine volume (mL/day)	1.2 ± 0.2	15.9 ± 0.8*	16.0 ± 1.8	17.6 ± 1.5	20.6 ± 1.3	21.9 ± 1.2#	6.0 ± 0.8#	3.0 ± 0.7#	2.1 ± 1.1#	11.1 ± 1.2#$
Urinary glucose excretion (mg/day)	0.9 ± 0.2	1,200 ± 190*	1,350 ± 60	2,130 ± 120#	2,560 ± 230#	3,630 ± 110#	929 ± 86	558 ± 113#	371 ± 145#	1990 ± 210#$

Note

NEFAs: non‐esterified fatty acids. Ipragliflozin and/or pioglitazone was orally administered to diabetic mice with NASH for 4 weeks. Values indicate mean ± *SEM* for six animals per group.

* *p* < .05 versus normal group.

^#^ *p* < .05 versus vehicle group.

^$^ *p* < .05 versus pioglitazone (10 mg/kg) group.

In the OGTT performed at Week 3, ipragliflozin improved glucose tolerance and insulin resistance in a dose‐dependent manner, effects that were significant at doses of 1 mg/kg or higher (Figure 2). Pioglitazone also dose‐dependently and significantly improved these parameters at doses of 10 mg/kg or higher, indicating that both drugs had similar efficacy for these outcomes. Combined treatment comprising ipragliflozin and pioglitazone also significantly and additively improved glucose tolerance and insulin resistance.

Following repeated administration for 4 weeks, ipragliflozin had significantly raised urinary glucose excretion and the associated urine volume, and decreased nonfasting blood glucose, HbA_1c_, and insulin levels in a dose‐dependent manner (Table 1). Ipragliflozin also significantly reduced body and visceral fat weights without any substantial effects on food consumption throughout the study period. Plasma lipid (triglycerides, non‐esterified fatty acids [NEFAs], and cholesterol) levels were also significantly reduced. Similarly, pioglitazone had significantly decreased nonfasting blood glucose, HbA_1c_, insulin, and lipid parameter levels in a dose‐dependent manner. Unlike ipragliflozin, pioglitazone decreased urinary glucose excretion via attenuation of hyperglycemia. Furthermore, pioglitazone significantly increased body and visceral fat weights without affecting food intake. Compared to the pioglitazone‐treated group, combined treatment comprising ipragliflozin and pioglitazone significantly raised urinary glucose excretion and the associated urine volume, and additively reduced nonfasting blood glucose, HbA_1c_, plasma insulin, and lipid parameter levels. Furthermore, body and visceral fat weights were significantly decreased without effects on food consumption.

**Figure 3:**
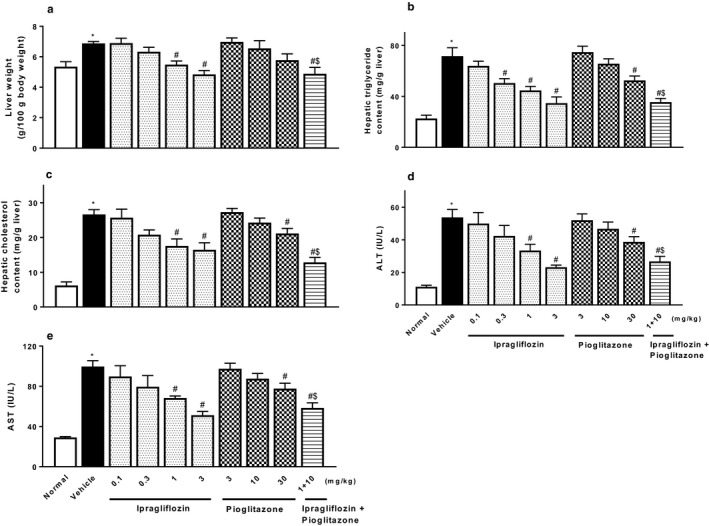
Effects of single or combined treatment with ipragliflozin and pioglitazone on NASH‐related parameters: (a) liver weight, hepatic contents of (b) triglycerides and (c) cholesterol, and plasma levels of (d) ALT and (e) AST, in type 2 diabetic mice with NASH. Ipragliflozin and/or pioglitazone was orally administered to diabetic mice with NASH for 4 weeks. Values indicate mean ± *SEM* for six animals per group. *p < .05 versus normal group, ^#^ *p* < .05 versus vehicle group, ^$^ *p* < .05 versus pioglitazone (10 mg/kg) group

**Figure 4:**
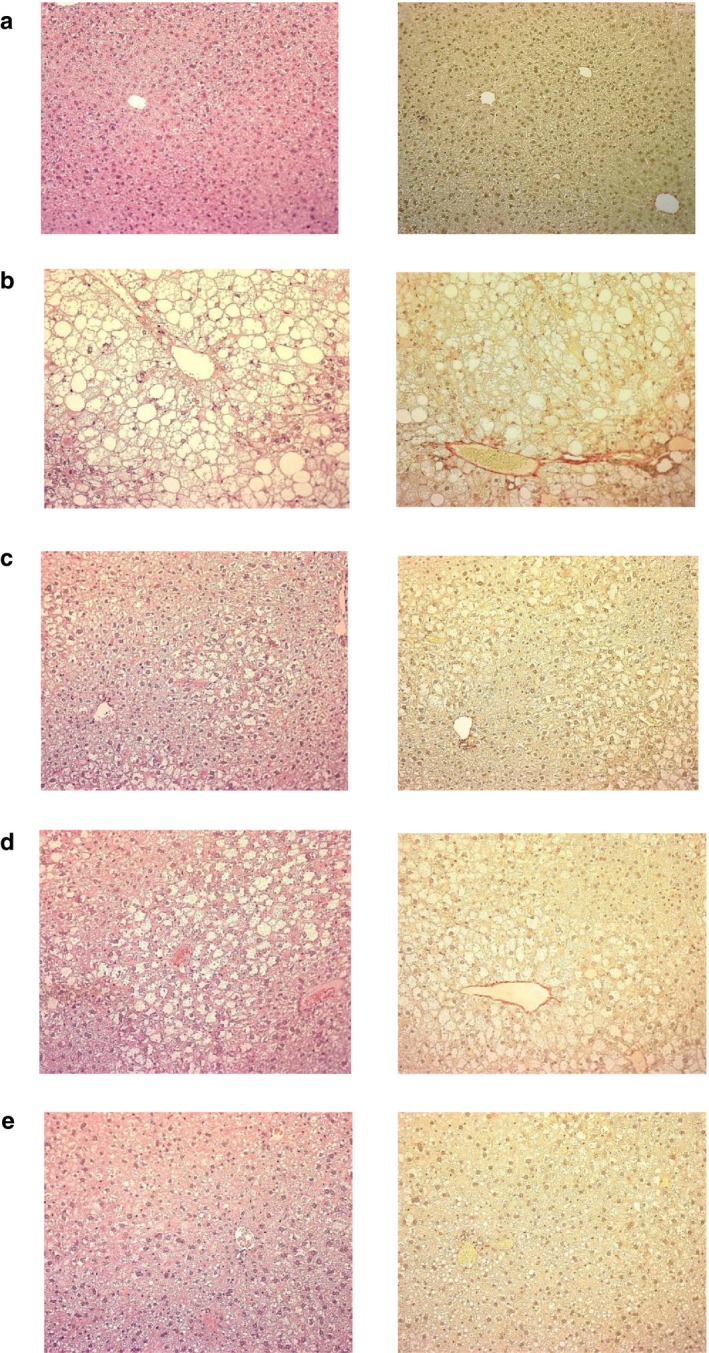
Representative light micrographs of liver tissue obtained from (a) normal, (b) vehicle‐treated, (c) ipragliflozin (1 mg/kg)‐treated, (d) pioglitazone (10 mg/kg)‐treated, and (e) ipragliflozin (1 mg/kg) + pioglitazone (10 mg/kg)‐treated type 2 diabetic mice with NASH. Ipragliflozin and/or pioglitazone was orally administered to diabetic mice with NASH for 4 weeks. The tissues were stained with hematoxylin and eosin (left) and sirius red (right). Magnification: ×100

**Figure 5:**
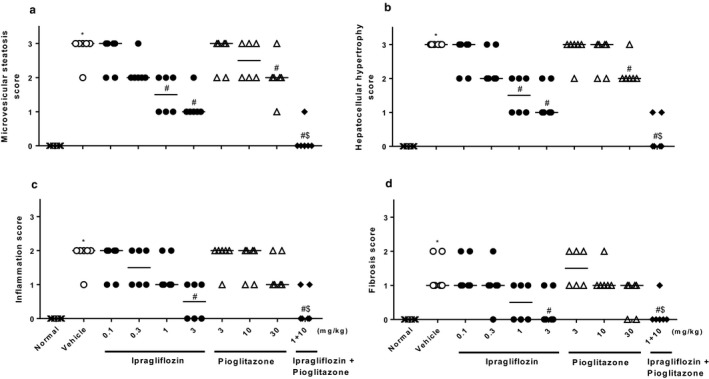
Effects of single or combined treatment with ipragliflozin and pioglitazone on liver histopathological scores for (a) microvesicular steatosis, (b) hepatocellular hypertrophy, (c) inflammation, and (d) fibrosis in type 2 diabetic mice with NASH. Ipragliflozin and/or pioglitazone was orally administered to diabetic mice with NASH for 4 weeks. Values indicate individual data and median for six animals per group. *p < .05 versus normal group, ^#^ *p* < .05 versus vehicle group, ^$^ *p* < .05 versus pioglitazone (10 mg/kg) group

Ipragliflozin significantly decreased liver weight, hepatic lipid contents, and plasma levels of ALT/AST in a dose‐dependent manner (Figure 3). Pioglitazone also significantly or showed a trend toward significantly reducing these steatohepatitis parameters, and combined treatment comprising ipragliflozin and pioglitazone significantly and additively reduced these parameters. Histopathologically, type 2 diabetic mice with NASH showed marked hepatocellular hypertrophy, microvesicular steatosis with focal lymphocytic infiltration, moderate lobular inflammation, and mild fibrosis (Figures 4 and 5). Ipragliflozin significantly improved these liver injury features including fibrosis in a dose‐dependent manner. Pioglitazone also significantly or showed a trend toward significantly reducing these liver injury features. The effects of pioglitazone were weaker than those of ipragliflozin. Combined treatment comprising ipragliflozin and pioglitazone significantly and additively improved these injury features.

**Figure 6:**
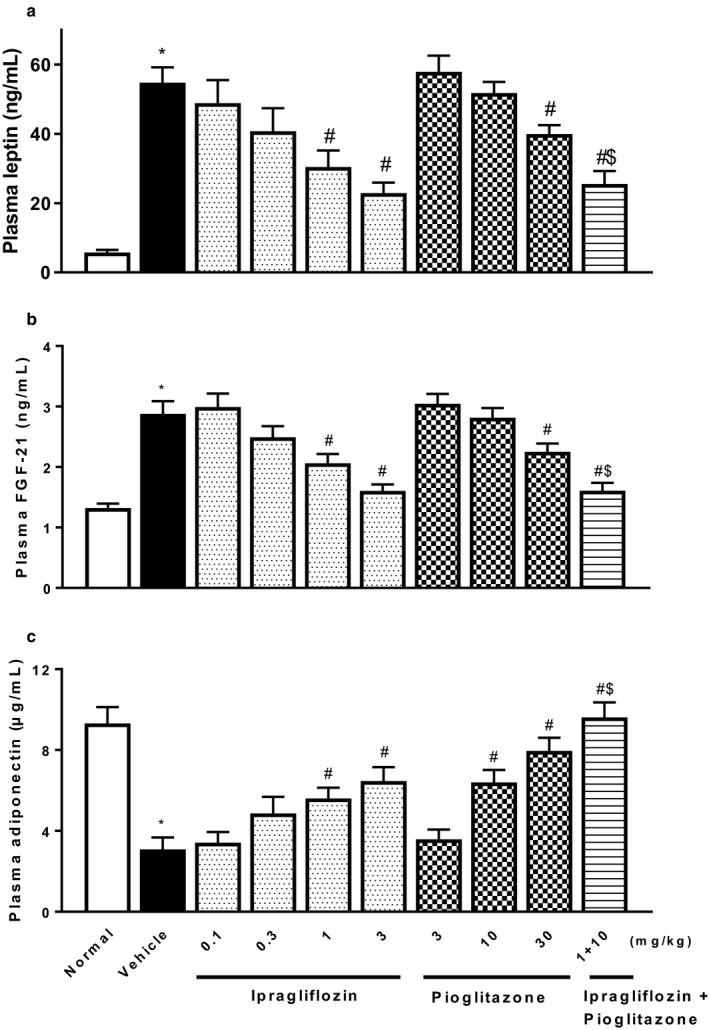
Effects of single or combined treatment with ipragliflozin and pioglitazone on plasma levels of (a) leptin, (b) FGF‐21, and (c) adiponectin in type 2 diabetic mice with NASH. Ipragliflozin and/or pioglitazone was orally administered to diabetic mice with NASH for 4 weeks. Values indicate mean ± *SEM* for six animals per group. *p < .05 versus normal group, ^#^ *p* < .05 versus vehicle group, ^$^ *p* < .05 versus pioglitazone (10 mg/kg) group

**Figure 7:**
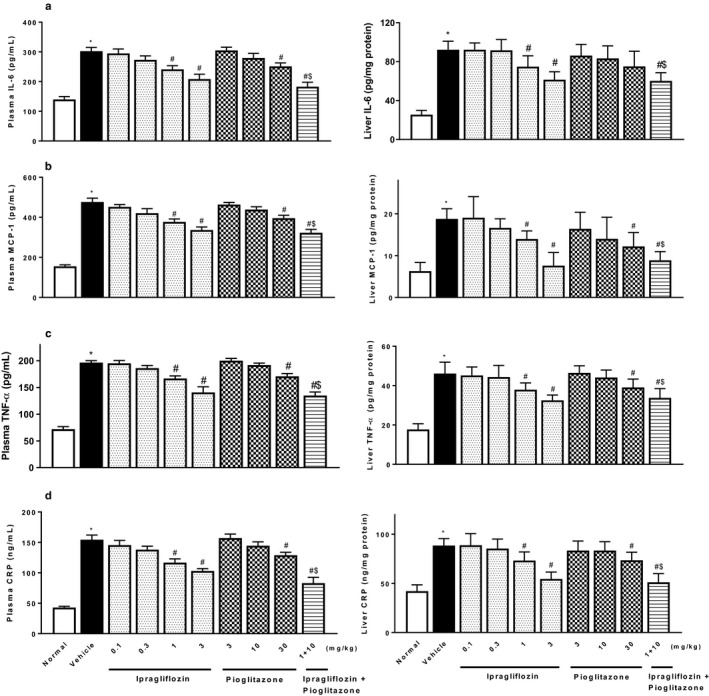
Effects of single or combined treatment with ipragliflozin and pioglitazone on plasma (left) and hepatic (right) levels of (a) IL‐6, (b) MCP‐1, (c) TNF‐α, and (d) CRP in type 2 diabetic mice with NASH. Ipragliflozin and/or pioglitazone was orally administered to diabetic mice with NASH for 4 weeks. Values indicate mean ± *SEM* for six animals per group. *p < .05 versus normal group, ^#^ *p* < .05 versus vehicle group, ^$^ *p* < .05 versus pioglitazone (10 mg/kg) group

**Figure 8:**
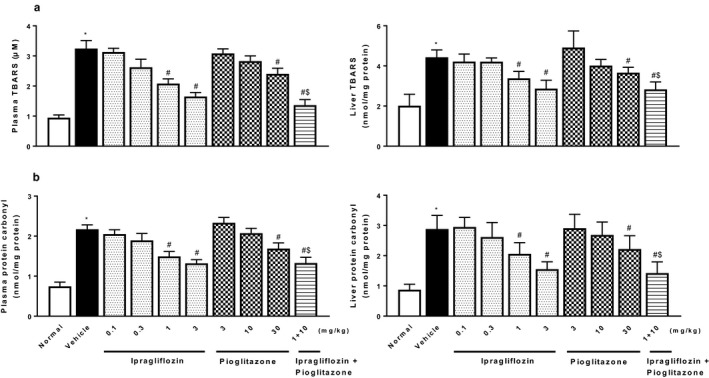
Effects of single or combined treatment with ipragliflozin and pioglitazone on oxidative stress parameters, plasma (left) and hepatic (right) levels of (a) TBARS and (b) protein carbonyl, in type 2 diabetic mice with NASH. Ipragliflozin and/or pioglitazone was orally administered to diabetic mice with NASH for 4 weeks. Values indicate mean ± *SEM* for six animals per group. *p < .05 versus normal group, ^#^ *p* < .05 versus vehicle group, ^$^ *p* < .05 versus pioglitazone (10 mg/kg) group

Type 2 diabetic mice with NASH showed significant changes in plasma levels of adipocytokines, increases in leptin and FGF‐21, and decreases in adiponectin (Figure 6). Ipragliflozin and pioglitazone significantly reversed the changes in plasma levels of these adipocytokines. Furthermore, type 2 diabetic mice with NASH showed significant increases in plasma and liver levels of proinflammatory cytokines (IL‐6, MCP‐1, and TNF‐α), the inflammatory parameter CRP (Figure 7), and oxidative stress biomarkers (TBARS and protein carbonyl) (Figure 8). Ipragliflozin and pioglitazone significantly reduced, or showed a trend toward reducing levels of these inflammatory and oxidative stress markers in a dose‐dependent manner, and combined treatment comprising ipragliflozin and pioglitazone also significantly and additively decreased these parameters.

## 4 DISCUSSION

NASH constitutes a major health concern, with a prevalence of up to 30% in the general population, and can be link to the increasing incidence of obesity and type 2 diabetes caused by elevated consumption of high‐fat and carbohydrate diets and reduced exercise across much of the industrialized world (Patton et al., 2006). Despite the high unmet needs and current recommendations of lifestyle modification being only partially effective, there are currently no approved drugs for the treatment of NASH (Bradford, Dillon, & Miller, 2014; Dyson, Anstee, & McPherson, 2015). To facilitate the development of novel therapies for NASH, pharmacological experiments should be performed in animal models with similar pathophysiology to those observed in human NASH. Animal models of NASH can be roughly categorized into those caused by genetic mutation and those with an acquired phenotype induced via dietary manipulation. Genetic leptin‐deficient (ob/ob), leptin‐resistant (db/db), or Agouti mutation (KK/A^y^) mice, and methionine/choline‐deficient (MCD) diet, CDAA diet‐, or high‐fat diet‐fed models (Anstee & Goldin, 2006; Ibrahim, Hirsova, Malhi, & Gores, 2016; Nakamura & Terauchi, 2013) are examples of animal models of NASH that have been used to examine a number of traditional and novel agents to date (Hansen et al., 2017). We previously examined the effects of ipragliflozin on NAFLD and NASH using CDAA diet‐fed rats, KK/A^y^, high‐fat diet‐fed streptozotocin–nicotinamide‐induced type 2 diabetic mice (Hayashizaki‐Someya et al., 2015; Tahara et al., 2013; Tahara, Takasu, Yokono, Imamura, & Kurosaki, 2016). NASH is thought to be the hepatic manifestation of metabolic syndrome due to its link to insulin resistance and obesity. Moreover, of the histological features of NASH, hepatic fibrosis has the strongest correlation with end‐stage liver disease and mortality (Takaki, Kawai, & Yamamoto, 2013). While the above‐mentioned animal models exhibit several pathophysiological abnormalities observed in human NASH, including hepatic steatosis, they are lacking in that they do not exhibit insulin resistance or liver fibrosis. This causes inaccurate translation of results obtained from these nonclinical models to clinical settings and the resulting development of therapies for NASH. The most suitable animal model of NASH should exhibit metabolic abnormalities, including insulin resistance and obesity, as well as liver histology that includes steatosis and fibrosis. A recent study reported that dietary manipulations could be used to progress steatosis to fibrosing steatohepatitis in type 2 diabetic mice (Okumura et al., 2005). According to this report, we developed a new animal model of NASH by applying dietary manipulation to a genetic type 2 diabetes model to induce more severe fibrosing steatohepatitis.

In addition to steatosis, this mouse model exhibited inflammation and fibrosis in the liver, along with type 2 diabetic features such as hyperglycemia, insulin resistance, lipid abnormalities, and obesity. Therefore, a major strength of this type 2 diabetes with NASH model is that it exhibits many of the biochemical and pathological characteristics observed in human NASH. We examined the effects of ipragliflozin and pioglitazone using these mice. Once‐daily administration of ipragliflozin caused long‐lasting reductions in blood glucose and plasma insulin levels. These potent and prolonged pharmacological effects of ipragliflozin are consistent with its wide distribution and prolonged retention in the target organ, the kidneys, shown in a pharmacokinetics experiment, and the persistent increase in urinary glucose excretion, shown in a pharmacodynamics experiment in a previous study (Tahara et al., 2016). Repeated administration of ipragliflozin lowered HbA_1c_ levels by increasing urinary glucose excretion. Further, ipragliflozin improved glucose tolerance, hyperinsulinemia, and insulin resistance, similar to findings reported in previous studies in type 2 diabetes models (Tahara et al., 2013, 2017; Tahara, Takasu, Yokono, Imamura, & Kurosaki, 2016). Additionally, no hypoglycemic symptoms such as excessive weight loss or decreased locomotor activity were noted throughout the present study period. Likewise, no risk of acute kidney failure was observed, as assessed using several parameters including changes in plasma creatinine levels induced by volume depletion via an increase in urine volume (data not shown). These results demonstrate that ipragliflozin is similarly useful for treating diabetic features in this type 2 diabetes with NASH model. Ipragliflozin also improved hyperlipidemia and reduced visceral fat and body weights without affecting food intake. These obesity‐improving effects of ipragliflozin may be due to the calorie loss induced by urinary glucose excretion. In addition, ipragliflozin reportedly reduces body fat mass by increasing lipolysis and fatty acid oxidation but not lean mass or bone mineral content in high‐fat diet‐induced obese rats (Yokono et al., 2014), and reverses abnormal regulation of appetite‐regulating hormones, such as neuropeptide Y and ghrelin, which are strongly correlated with obesity and lipid abnormalities (Tahara, Kondo, Takasu, & Tomiyama, 2018). Abnormal lipid metabolism and obesity are causative environmental factors for the development of various metabolic diseases including type 2 diabetes (Horton, 2009). These findings suggest that ipragliflozin has the therapeutic potential to improve lipid abnormalities and obesity in type 2 diabetes with NASH.

We found that ipragliflozin significantly decreased plasma aminotransferase levels and ameliorated hepatic steatosis as well as inflammation and fibrosis in the type 2 diabetes with NASH mouse model. The occurrence of NASH is strongly associated with insulin resistance and other metabolic abnormalities including type 2 diabetes, obesity, hyperlipidemia, and hypertension. Evidence indicates that there is a bidirectional relationship between NASH and insulin resistance: insulin resistance enhances hepatic steatosis by elevating free fatty acid flux from adipose tissue and uptake by the liver, impaired fatty acid oxidation, and higher de novo hepatic lipogenesis (Neuschwander‐Tetri, 2010), which causes impaired glucose metabolism and insulin resistance, generating a vicious cycle (Bugianesi, McCullough, & Marchesini, 2005). Our findings suggest that as a result of elevating urinary glucose excretion, ipragliflozin improves insulin resistance and other metabolic abnormalities such as hyperglycemia and dyslipidemia. Furthermore, ipragliflozin reduces obesity by producing a negative energy balance. Evidence from a large number of studies indicates that consumption of too much dietary carbohydrate raises free fatty acid delivery to the liver, inadequate fatty acid oxidation, and de novo lipogenesis. Therefore, reducing carbohydrate consumption and obesity using a calorie‐restricted diet and physical exercise can reduce the incidence of insulin resistance and metabolic syndrome and resolve hepatic steatosis, and should form part of any NASH treatment regimen (Bradford et al., 2014). The anti‐diabetic and anti‐obesity effects of ipragliflozin may contribute to improving NASH in a similar manner.

A two‐hit theory has been proposed to explain the development of NASH (Takaki et al., 2013). The first hit is the above‐mentioned hepatic steatosis and the second hit includes cellular stresses such as oxidative stress, apoptosis, and inflammation. Among these, increased plasma levels of proinflammatory cytokines such as TNF‐α and IL‐6 are common findings in NASH patients (Wu et al., 2010). Abnormally high plasma levels of proinflammatory cytokines have been reported among type 2 diabetes patients with NAFLD, which may be induced by several conditions, including hyperglycemia, insulin resistance, and obesity, causing impaired insulin signaling, cell damage, neutrophil chemotaxis, hepatic stellate cell activation, and apoptosis (Shams, Al‐Gayyar, & Barakat, 2011). These could potentially exacerbate systemic and hepatic inflammation and fibrosis. Furthermore, oxidative stress is known to lead to cell and tissue damage via the production of reactive oxygen species such as active oxygen and free radicals. This oxidative stress, mainly caused by mitochondrial dysfunction, is thought to play an important role in the progression of liver damage in NASH (Sumida, Niki, Naito, & Yoshikawa, 2013). A previous study reported increased oxidative stress among type 2 diabetes patients with NAFLD (Shams et al., 2011), which can be mainly explained by hyperglycemia and other mechanisms such as glucose autoxidation, increased nitric oxide synthase activity, and free fatty acid oxidation (Baskol, Baskol, & Kocer, 2007). In this study, type 2 diabetic mice with NASH exhibited significantly elevated plasma and liver levels of proinflammatory cytokines and oxidative stress biomarkers, which are thought to play an important role in the progression of liver fibrosis in NASH (Rolo, Teodoro, & Palmeira, 2012), and ipragliflozin potently attenuated these increases. This indicates that ipragliflozin is not only beneficial because it ameliorates hepatic steatosis but also because it reduces inflammation, oxidative stress, and fibrosis in the type 2 diabetes with NASH mouse model. In the present study, liver injury was estimated using a simple scoring method on microscopic autopsy. Additional detailed investigations, such as those using the NAFLD activity score or quantitative image analysis of collagen in the liver, should be performed to confirm the pharmacologic effects of ipragliflozin on liver fibrosis in NASH.

Pioglitazone, a thiazolidinedione derivative, is an anti‐diabetic (insulin‐sensitizing) agent and selective agonist for peroxisome proliferator‐activated receptor (PPAR)‐γ, a transcription factor that regulates gene expression in the liver, adipose, vascular endothelium, and muscle tissue, and improves insulin action, glucose metabolism, inflammation, and adipocyte biology in type 2 diabetic patients with insulin resistance (Chiarelli & Di Marzio, 2008). Most studies have examined the use of one of two classes of insulin‐sensitizing drugs on NASH: thiazolidinediones (pioglitazone and rosiglitazone) or biguanides (metformin), for improving insulin resistance as a possible treatment for NASH (Van Wagner & Rinella, 2011; Xu et al., 2016). Several clinical trials have reported the beneficial role of pioglitazone in patients with NASH (Bril et al., 2018). A recent meta‐analysis that evaluated five high‐quality randomized controlled trials, including the PIVENS study, concluded that pioglitazone significantly reduced serum levels of aminotransferases and improved necroinflammatory scores (ballooning, steatosis, and lobular inflammation), but did not improve hepatic fibrosis (Boettcher et al., 2012). Therefore, while pioglitazone attenuates hepatic steatosis and metabolic abnormalities including insulin resistance, and remains a promising drug for NASH treatment, it is unclear whether pioglitazone ameliorates hepatic fibrosis in NASH.

In this study, pioglitazone significantly reduced blood glucose and plasma insulin levels. Repeated administration of pioglitazone not only reduced HbA_1c_ levels but also improved glucose tolerance, hyperinsulinemia, and insulin resistance. Thiazolidinediones via their agonist activity on PPAR‐γ promote the uptake of circulating fatty acids into adipocytes. The glucose‐lowering effects of thiazolidinediones are due to increased disposal of glucose into adipose tissues along with increased expression of insulin sensitizing factors, such as adiponectin (Hirose et al., 2002), and decreased expression of proteins that promote insulin resistance such as FGF‐21 (Li et al., 2009). Studies have confirmed that treatment with pioglitazone in NASH is closely associated with an increase in plasma adiponectin levels and improved insulin sensitivity in adipose tissue, liver, and skeletal muscle (Cusi, 2012). In this study, pioglitazone significantly reversed plasma levels of adipocytokines including adiponectin, which is consistent with findings from clinical studies. Therefore, pioglitazone prevents the progression of type 2 diabetes, an effect that may be strongly linked to its ability to improve dysregulation of adipocytokines. Repeated administration of pioglitazone significantly improved hyperlipidemia, but increased visceral fat and body weights without affecting food intake, which is consistent with findings from clinical studies on type 2 diabetes (Aghamohammadzadeh et al., 2015; Ito et al., 2017). Increased fat and body weights during pioglitazone treatment has been attributed to promotion of fatty acid storage and uptake in adipose tissue via the regulation of a number of genes with roles in fatty acid uptake (fatty acid transport protein 1, adipocyte fatty acid‐binding protein, and CD36) and adipogenesis (sterol regulatory element‐binding protein‐1 and stearoyl‐CoA desaturase 1) (Wu et al., 2010). Despite increasing fat and body weights, pioglitazone potently improved diabetic abnormalities including hyperlipidemia via these mechanisms in type 2 diabetes.

In the present study, pioglitazone significantly reduced plasma aminotransferase levels and ameliorated hepatic steatosis. Furthermore, pioglitazone significantly or showed a trend toward significantly attenuating inflammation, oxidative stress, and pathological injuries in the liver. As mentioned above, pioglitazone enhances the redistribution of fat from several tissues including liver to adipocytes. This increased adipocyte fat mass suggests that attenuation of hepatic steatosis may be a consequence of the diversion of fat storage from the liver to adipose tissue via stimulation of PPAR‐γ in adipocytes (Wu et al., 2010). Pioglitazone significantly attenuated plasma and hepatic inflammation and oxidative stress in type 2 diabetic mice with NASH. Consistent with present findings, PPAR‐γ agonists reportedly inhibit proinflammatory cytokines and oxidative stress in the liver of NASH mice (Wu et al., 2010), which may lead to improved insulin resistance, hepatic lipid metabolism, and liver injury. Furthermore, this mechanism of action may also be involved in reversing activated hepatic stellate cells into a quiescent phenotype and reducing the expression of TGF‐β1, a master regulator of fibrosis (Kawaguchi et al., 2004; Nan et al., 2009).

Combined treatment comprising ipragliflozin and pioglitazone significantly and additively improved diabetic features and metabolic abnormalities in addition to NASH. Combination treatment with the two drugs additively reversed the changes in plasma levels of adipocytokines, suggesting that ipragliflozin and pioglitazone attenuate insulin resistance, at least in part, via different mechanisms such as direct anti‐hyperglycemic effects and insulin‐sensitizing activity in the liver, adipose, and muscle tissues. Several studies have reported the beneficial effects of coadministration of SGLT2 inhibitors with PPAR‐γ agonists in type 2 diabetes. Combined treatment with the SGLT2 inhibitor canagliflozin and pioglitazone additively improved insulin resistance in type 2 diabetic mice (Watanabe et al., 2015). We previously reported that combined treatment with ipragliflozin and pioglitazone additively improved hyperglycemia in type 2 diabetic mice (Takasu, Hayashizaki, Tahara, Kurosaki, & Takakura, 2015). In a clinical experiment, combination therapy with the SGLT2 inhibitor dapagliflozin attenuated hyperglycemia in patients with type 2 diabetes that was inadequately controlled using pioglitazone monotherapy (Rosenstock, Vico, Wei, Salsali, & List, 2012). In addition, combining dapagliflozin with pioglitazone additively attenuated progression of nephropathy in type 2 diabetes (Han et al., 2018). The present study demonstrated that combined treatment comprising ipragliflozin and pioglitazone additively attenuated NASH. We investigated the additive effects of the two drugs using a submaximal dose of each drug. Detailed additive effects using a maximal dose of each drug should be performed to confirm the combinatory effects of ipragliflozin and pioglitazone on NASH. In addition, while this type 2 diabetes with NASH model exhibited many of the biochemical and pathological characteristics observed in human NASH, it also showed several different characteristics such as severe hyperglycemia. Additional detailed examinations using other animal models are needed. Despite these limitations, the present study demonstrated, for the first time, the possibility that combination therapy comprising ipragliflozin and pioglitazone can additively prevent the development of NASH.

As mentioned above, in this type 2 diabetes with NASH model, pioglitazone significantly attenuated microvesicular steatosis and hepatocellular hypertrophy. In contrast, hepatic inflammation and fibrosis tended to improve, but these effects were weak and not significant. Nonclinical studies have shown that pioglitazone significantly improves hepatic steatosis and inflammation and fibrosis (Kawaguchi et al., 2004; Nan et al., 2009). In contrast, clinical studies have shown that pioglitazone improves plasma aminotransferase levels and hepatic steatosis but not fibrosis (Boettcher et al., 2012; Van Wagner & Rinella, 2011). The discrepancy between nonclinical and clinical studies may be attributed to differences in the pathogenesis, genotype, and confounding factors of NASH between animal models and human patients. Additionally, pioglitazone may have the potential to attenuate hepatic pathological injuries including fibrosis, but the efficacy may be too weak to produce definitive pharmacological effects. Furthermore, pharmacotherapy comprising thiazolidinediones for fibrosis in NASH may necessitate long‐term treatment. However, such long‐term treatment is associated with problems with efficiency and safety, such as the occurrence of side effects including edema, cardiovascular disease, congestive heart failure, bladder cancer, and bone loss (Barnett, 2009). Further studies are warranted to clarify these issues. In contrast, ipragliflozin significantly reduced steatosis as well as fibrosis in this study, suggesting that SGLT2 inhibitors may be useful for the treatment of NASH. Furthermore, combined treatment comprising ipragliflozin and pioglitazone may be an excellent therapeutic option for type 2 diabetes and NASH.

In summary, this animal model recapitulates a number of phenotypes of NASH including hepatic steatosis and fibrosis and their associated metabolic features such as insulin resistance and obesity. We expect that this model will be useful for identifying pharmacologic targets/agents, progression mechanisms, and therapeutic approaches for NASH. Ipragliflozin reduced type 2 diabetic features including hyperglycemia, obesity, and insulin resistance and the pathophysiological progression of NASH. This suggests that the SGLT2 inhibitor ipragliflozin is not only beneficial for ameliorating hepatic steatosis but also reducing inflammation and fibrosis. Furthermore, combined treatment comprising ipragliflozin and pioglitazone additively improved these therapeutic effects. Therefore, ipragliflozin in monotherapy or coadministered with pioglitazone may attenuate development of the pathological features of NASH, suggesting its potential as a new therapeutic strategy for the treatment of NASH associated with type 2 diabetes. We anticipate that future clinical studies will demonstrate the usefulness of SGLT2 inhibitors in the treatment of NASH.

## CONFLICT OF INTEREST

The authors have no conflict of interest other than being employees of Astellas Pharma Inc.

## AUTHOR CONTRIBUTIONS

A.T. conceived and designed the research, performed the experiments, analyzed the data, interpreted the results of the experiments, and prepared figures and drafted the manuscript; A.T. and T.T. edited and revised the manuscript; A.T. approved the final version of the manuscript.
